# Editorial: Mendelian Randomization: Approach and Applications

**DOI:** 10.3389/fgene.2021.752146

**Published:** 2021-09-28

**Authors:** Guo-Liang Wu, Yao-Zhong Liu, Lei Zhang

**Affiliations:** ^1^Department of General Surgery, Suzhou Ninth Hospital Affiliated to Soochow University, Suzhou, China; ^2^Department of Biostatistics and Data Science, Tulane University School of Public Health and Tropical Medicine, New Orleans, LA, United States; ^3^Center for Genetic Epidemiology and Genomics, School of Public Health, Medical College of Soochow University, Suzhou, China

**Keywords:** Mendelian randomization, causal inference, horizontal pleiotropy, genome-wide association study, confounding factor

Mendelian randomization (MR) is a valuable approach to assess potential causal relationships between exposures and outcomes in an observational study, especially when traditional randomized controlled trials or observational studies are not feasible (Davey Smith and Ebrahim, 2003). Genetic variants, such as single-nucleotide polymorphism (SNP), are selected as instrumental variables due to random allocation at birth, which can effectively address unmeasured confounding bias and reverse causation. The productive findings of published genome-wide association studies (GWAS) for screening suitable instrumental variables of various exposure traits contributes to MR's increasing popularity as a major approach to infer potential causal associations (Sekula et al., 2016). However, the endeavor of MR is still limited in methodological development and empirical application.

Although MR analysis provides a time- and cost-efficient solution to infer causal relationships without additional recruitment or experimental design, certain threats, especially pleiotropy, weak instrument, and linkage disequilibrium, could violate core assumptions, leading to biased causal links (Burgess et al., 2015; Lawlor, 2016). Hence, it is urgent to develop and apply novel MR methodologies and assess existing methods to address those issues. Moreover, the application of MR has also extended to new data types and scenarios, such as mRNA, protein, metabolic biomarkers, molecular phenotypes, and microbiome. All these applications will further explore the etiological mechanisms behind human diseases.

Here, we organized a Research Topic on “*Mendelian Randomization: Approach and Applications*” which gathered a collection of 14 high-quality studies made up of 13 original articles and one review that deal with approaches and applications of MR. These studies emphasize on novel methodological development, comparison of existing statistical methods, causal inference between traditional traits/diseases or novel data types, and drug target application.

Five contributions in this issue clarified whether there is a causal relationship between traditional traits/diseases and identified their potential risk factors. Zhu et al. utilized a MR study to fill the gap on causal links between alcohol use and mental health in East Asian populations. Their study reported that alcohol consumption was causally associated with a lower risk of depression. The study by Cui, Hou, et al. employed the bidirectional causal association between inflammatory bowel disease and Ankylosing Spondylitis with a two-sample MR based on GWAS summary statistics. It indicated that inflammatory bowel disease was the causal factor of an increased risk of Ankylosing Spondylitis. Similarly, Gao X. et al. investigated the causal link between sleep-related phenotypes and type 2 diabetes mellitus and showed an adverse effect of insomnia on type 2 diabetes mellitus. On the other hand, evidence from the MR approach did not support a causal relation between certain associated conditions, which possibly represented other confounders in previous observational studies, as shown in two articles by Yang et al. and Cui, Feng, et al., respectively.

Six contributions in this issue explored possibilities in using new data types in MR analysis and application of MR in new scenarios. Zhang et al. utilized summary statistics of the gut microbiome to assess causal relationships in inflammatory bowel disease, which is a novel attempt to identify specific pathogenic bacteria taxa for complex diseases by MR. In a similar vein, Ha et al. examined the causal effects of 69 environmental factors on asthma in the MR analysis and found that body mass index causally affects the development of asthma. For protein level, Zheng et al. demonstrated that fibroblast growth factor 23 level was significantly and causally associated with large-artery atherosclerotic stroke, which offers potential therapeutic targets for the disease. Yet Wang B. et al. did not find evidence to support the causal relationship of C-reactive protein and fibrinogen with an increased risk of intracerebral hemorrhage. Moreover, Gao Y. et al. applied the genetic risk score and MR framework to assess the causality of leukocyte telomere length on reviewed around 100 MR studies of different types of exposures with risk of stroke and provided perspectives to reviewed around 100 MR studies of different types of exposures with risk of stroke and provided perspectives to future novel approaches, including drug development and repurposing.

Although the application of MR has become more popular, existing MR statistical methods still cannot completely solve the issues that violate the core assumptions, such as pleiotropy and linkage disequilibrium. Thereby, novel methodological development is warranted. In particular, three contributions have showed novel statistical methods and compared studies to optimize MR methods and address existing problems. The study by Schooling et al. has demonstrated theoretically and empirically that multivariable MR may effectively mitigate selection bias due to survival before recruitment and performed an example simulation after amelioration. Wang Y. et al. proposed a novel mixed-effects regression model-based method, Pleiotropic and linkage disequilibrium adaptive Mendelian randomization (PLDMR), which corrected linkage disequilibrium and pleiotropic effect in the causal statistical inference. The simulation results showed the validity and advantage of PLDMR compared with others. Finally, the contribution by Lin et al. provided an improved MR approach, Mendelian Randomization with Refined Instrumental Variable from Genetic Score (MR-RIVER), to integrate summary data of multiple instrumental variables into a single genetic score. Through statistical simulations, it indicated that this novel approach possessed more statistical power as well as smaller biases and mean squared errors than the competing methods.

In summary, articles in this issue have comprehensively illustrated that MR framework is an effective and powerful solution to causal inference in various application scenarios. We hope that our special issue will stimulate further research and promote the development of more efficient and accurate MR approaches in the near future.

## Author Contributions

Y-ZL and LZ conducted this topic issue. G-LW, LZ, and Y-ZL wrote the manuscript. All authors contributed to the article and approved the submission.

## Funding

LZ was partially supported by a project funded by the Priority Academic Program Development (PAPD) of Jiangsu higher education institutions.

## Conflict of Interest

The authors declare that the research was conducted in the absence of any commercial or financial relationships that could be construed as a potential conflict of interest.

## Publisher's Note

All claims expressed in this article are solely those of the authors and do not necessarily represent those of their affiliated organizations, or those of the publisher, the editors and the reviewers. Any product that may be evaluated in this article, or claim that may be made by its manufacturer, is not guaranteed or endorsed by the publisher.
